# Does inactivation of USP14 enhance degradation of proteasomal substrates that are associated with neurodegenerative diseases?

**DOI:** 10.12688/f1000research.7800.2

**Published:** 2016-04-25

**Authors:** Daniel Ortuno, Holly J. Carlisle, Silke Miller

**Affiliations:** 1 Department of Neuroscience, Amgen Inc., Thousand Oaks, CA, USA; 2 Department of Neuroscience, Amgen Inc., Cambridge, MA, USA

**Keywords:** Neurodegeneration, ubiquitin, proteasome, deubiquitinating enzyme, tau, TDP-43, ubiquitin-specific peptidase 14, protein clearance

## Abstract

A common pathological hallmark of age-related neurodegenerative diseases is the intracellular accumulation of protein aggregates such as α-synuclein in Parkinson’s disease, TDP-43 in ALS, and tau in Alzheimer’s disease. Enhancing intracellular clearance of aggregation-prone proteins is a plausible strategy for slowing progression of neurodegenerative diseases and there is great interest in identifying molecular targets that control protein turnover. One of the main routes for protein degradation is through the proteasome, a multisubunit protease that degrades proteins that have been tagged with a polyubiquitin chain by ubiquitin activating and conjugating enzymes. Published data from cellular models indicate that Ubiquitin-specific protease 14 (USP14), a deubiquitinating enzyme (DUB), slows the degradation of tau and TDP-43 by the proteasome and that an inhibitor of USP14 increases the degradation of these substrates. We conducted similar experiments designed to evaluate tau, TDP-43, or α-synuclein levels in cells after overexpressing USP14 or knocking down endogenous expression by siRNA.

## Introduction

Research on the ubiquitin-proteasome system has far reaching implications for the development of drugs to treat illnesses associated with the accumulation of misfolded proteins, including Alzheimer’s and Parkinson’s disease ( Ciechanover & Kwon, 2015). Ubiquitin-specific protease 14 (USP14), like its yeast ortholog Ubp6, is a proteasome-associated deubiquitinating enzyme (DUB) that is activated upon binding to the proteasome and catalyzes the cleavage of ubiquitin subunits from substrates before degradation by the proteasome ( Borodovsky *et al.*, 2001; Hanna *et al.*, 2006; Hu *et al.*, 2005). By releasing ubiquitin molecules from the substrate, USP14/Ubp6 helps to prevent the rapid degradation of ubiquitin molecules together with the substrate protein ( Hanna *et al.*, 2007). A critical role of USP14 in stabilizing cellular ubiquitin levels was demonstrated *in vivo* in USP14 deficient ax ^J^ mice which display decreased ubiquitin levels in all tissues with the greatest loss observed at synaptic terminals ( Anderson *et al.*, 2005; Wilson *et al.*, 2002).

In addition to maintaining cellular ubiquitin pools, USP14/Ubp6 has been shown to modulate substrate degradation. Goldberg and colleagues showed that upon binding to a substrate’s polyubiquitin chain, activated USP14/Ubp6 facilitates gate-opening of the proteasome ( Peth *et al.*, 2009). This mutual interaction of USP14/Ubp6 with the proteasome is thought to enhance selectivity of the proteasome for ubiquitinated proteins and couple deubiquitination to degradation. In contrast, Finley and colleagues found that USP14/Ubp6, and in some instances a catalytically inactive mutant (C114A in mammals), could cause an inhibition of the degradation of substrates ( Hanna *et al.*, 2006; Lee *et al.*, 2010). For model substrates and ataxin3, this effect was shown to require USP14/Ubp6 protein but not its catalytic activity. For two proteins involved in neurodegenerative diseases, tau and TDP-43, inhibition of proteasomal degradation by USP14 was dependent on its deubiquitinating activity, since the catalytically inactive mutant had no effect ( Lee *et al.*, 2010). This led to the hypothesis and proof thereof that deubiquitination of substrates by USP14 at a faster rate than the proteasome initiates degradation could cause rejection of otherwise competent substrates from the proteasome ( Lee *et al.*, 2016). Inhibition of USP14 by a small molecule inhibitor (IU1) enhanced proteasomal substrate degradation in cells overexpressing tau or TDP-43 ( Lee *et al.*, 2010). Thus, inhibition of USP14 was proposed as a therapeutic strategy to enhance proteasomal function in neurodegenerative diseases in which these proteins accumulate.

## Methods

*Constructs.* Human USP14 (hUSP14wt), V5-tagged hUSP14wt (V5-hUSP14wt), catalytically inactive mutant USP14-C114A (hUSP14CA), V5-tagged hUSP14CA (V5-hUSP14CA), human tau, and Flag-tagged human TDP- 43 (Flag-TDP-43) were cloned into the pTT5d expression vector by Amgen’s Protein Sciences department and confirmed by sequencing. Human α-synuclein-Flag CMV6 expression vector was purchased from Origene (#RC221446) and confirmed by sequencing.

*Cell lines.* All cell lines were obtained from ATCC. HEK293 cells were grown in DMEM/10% fetal bovine serum/1% penicillin, streptomycin, glutamine. U2OS cells stably expressing Flag-tagged human α-synuclein (U2OS/synuclein) were generated by Amgen Neuroscience in San Francisco and grown in McCoy’s 5A/10% fetal bovine serum/1% penicillin, streptomycin/2% glutamine and 0.5mg/mL G418. SH-SY5Y cells were grown DMEM/10% fetal bovine serum/1% penicillin, streptomycin, glutamine and 0.5mg/mL G418. All cells were grown in incubators at 5%CO _2_/37°C. All cell culture reagents were purchased from Gibco.

*Transfections.* HEK293 cells were plated at a density of 10 ^-6^ cells/well in 6-well plates and transfected with plasmids using Lipofectamine™ 2000 (Thermofisher) for 4 hours, and analyzed 48 hours after transfection. U2OS/synuclein cells were plated at 5×10 ^-4^ cells/well in 24-well plates and SH-SY5Y cells were plated at 2×10 ^-5^ cells/well in 6-well plates. Cells were transfected with Opti-MEM™ (Thermofisher) containing 100nM siRNA, and analyzed 60, 72 or 96 hours after transfection. USP14 siRNAs were obtained from Ambion.

*Western blot.* Cells were lysed with Lysis Reagent (Roche) containing 1% SDS/1X Complete™ protease inhibitors cocktail tablets (Roche). Samples were boiled and Benzonase Nuclease (Sigma) was added following the manufacturer’s instructions. 10ug of lysate was loaded on a 12% Bis-Tris gel (Life-Sciences) and proteins were separated by electrophoresis (100mA, 200V) and transferred onto 0.2µm nitrocellulose membrane (Life Sciences) for a minimum of 4hrs (100mA, 25V). Membranes were blocked with Odyssey Blocking Buffer (Li-Cor), incubated with primary antibodies diluted in Li-Cor buffer with 0.2% Tween-20 at 4°C shaking overnight, and washed 3× with phosphate-buffered saline/0.1% Tween-20 (PBST). Membranes were then incubated with secondary antibodies for 1 hour at room temperature in the dark, washed 3× with PBST, and analyzed with the Odyssey imaging system at a relative intensity setting of 2–2.5 for the 800 channel and 1–2 for the 700 channel. Beta-actin or GAPDH served as a loading control.

*Antibodies.* Mouse monoclonal anti-tau5 (1µg/ml; Invitrogen AHB0042), mouse monoclonal beta-actin (1:1000; Cell Signaling 3700S), mouse monoclonal anti-flag (1:500; Sigma-Aldrich F1804), mouse monoclonal anti-V5 (1µg/ml, Sigma-Aldrich V8012), mouse monoclonal anti-GAPDH (1µg/ml; Invitrogen 39–8600), chicken polyclonal anti-USP14 (5µg/ml; Lifesensors AB505), IRDye 680 or 800 anti-mouse or anti-chicken infrared secondary antibodies (1:10000; Li-Cor).

*Data analysis*. Ratios of the intensity readings for the protein of interest and the loading control were calculated in Microsoft Excel 2010 and plotted using GraphPad Prism 6.05.

## Results

**Figure 1.**
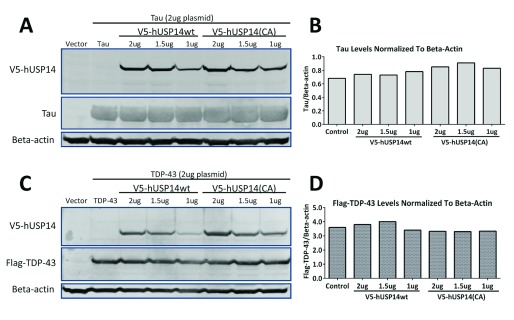
Tau and TDP-43 levels were not increased when coexpressed with V5-tagged hUSP14wt versus V5-tagged hUSP14(CA). 1, 1.5 or 2ug of V5-hUSP14wt (wt = wild type) or V5-hUSP14(CA) (CA = C114A, catalytically inactive) were cotransfected with 2ug Tau or Flag-TDP-43 plasmid in HEK293 cells. Cells were lysed after 48 hours and analyzed by western blot using a standard protocol. Actin served as loading control. Despite robust expression of USP14 or its catalytically inactive mutant as detected by the V5-tag ( **A**, **C**), no differences were observed in Tau ( **A**, **B**) or Flag-TDP-43 ( **C**, **D**) protein levels. Note that we did not observe differences in the expression levels of USP14 versus USP14(CA). Control = empty vector control.

A key experiment from Lee *et al.*, 2010, (Figure 1g) showed that recombinantly expressed tau or TDP-43 levels in HEK293 cells were higher when coexpressed with wild type as compared to catalytically inactive (C114A) USP14. We cotransfected V5-pTT5d-USP14 or V5-pTT5d-USP14 (C114A) plasmids (ranging from 0.5 to 2µg) and 2µg pTT5d-Tau or pTT5d-Flag-TDP-43 plasmids in HEK293 cells. Note that we used a pTT5d vector to express proteins, while Finley and colleagues used a pcDNA3.1 vector (Invitrogen). Despite robust expression of USP14 or the catalytically inactive mutant as detected by anti-V5 antibody ( Figure 1A, C), no decrease was observed in the levels of tau ( Figure 1A, B) or TDP-43 ( Figure 1C, D) in cells transfected with the catalytically inactive mutant compared to wild type USP14. A similar experiment was performed in which 1 µg of Tau or TDP-43 was cotransfected with 2 µg USP14 constructs for 48 hours and this also did not appear to alter Tau or TDP-43 levels (not shown).

**Figure 2.**
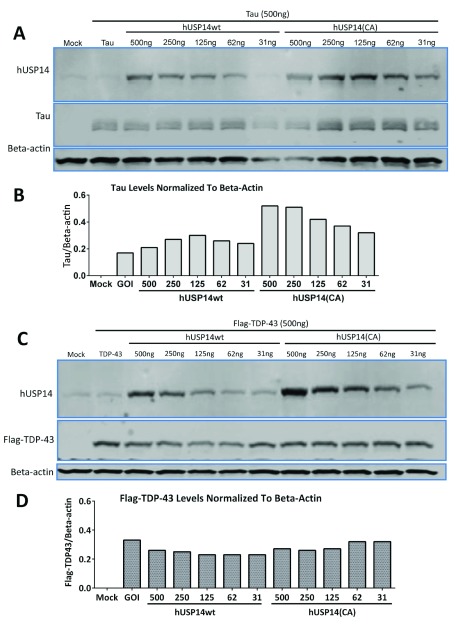
No decreases observed in tau or TDP-43 levels after cotransfection with untagged hUSP14(CA) versus untagged hUSP14wt. 31 to 500ng of hUSP14wt or hUSP14(CA) plasmids were cotransfected with 2ug tau or TDP-43 plasmid in HEK293 cells. Cells were lysed after 48 hours and analyzed by western blot using a standard protocol. Actin served as loading control. Despite robust expression of USP14 or the catalytically inactive mutant as detected by anti-USP14 antibody ( **A**, **C**), no decreases were observed in tau ( **A**, **B**) or TDP-43 ( **C**, **D**) protein levels in the cells transfected with hUSP14CA. Note that we did not observe differences in the expression levels of USP14 versus USP14(CA). Mock = empty vector control, GOI = gene of interest and refers to either tau or TDP-43 in the absence of USP14 cotransfection.

To exclude the possibility that the V5-tag rendered the USP14 constructs non-functional, we validated an anti-USP14 antibody ( Supplementary material) and tested untagged USP14 constructs in TDP-43 overexpressing cells. HEK293 cells were transfected with USP14 or USP14(C114A) plasmids at concentrations ranging from 31ng to 4µg and tau and Flag-TDP-43 at concentrations of 0.5µg; representative blots are shown in Figure 2. Despite robust expression of USP14 or its catalytically inactive mutant as detected by the USP14 antibody, no decrease was observed in tau or Flag-TDP-43 protein levels in cells transfected with the catalytically inactive mutant compared to wild type USP14 ( Figure 2A, B). Two similar experiments were conducted with 2 µg Tau or TDP-43 cotransfected with 0.05 or 0.1 µg USP14 constructs or 4 µg Tau or TDP-43 cotransfected with 0.5, 1.0, 2.0 or 4.0 µg of the USP14 constructs; Tau and TDP-43 levels did not appear altered in either experiment (not shown).

**Figure 3.**
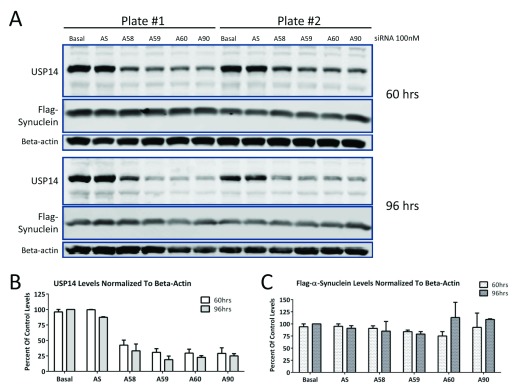
siRNA knockdown of endogenous USP14 does not decrease α-synuclein levels in U2OS cells stably expressing α-synuclein. U2OS cells stably expressing Flag-α-synuclein were treated with 100nM USP14 siRNA from Ambion (A58, A59, A60 or A90) for 60 or 96 hours ( **A**). Scrambled siRNA (AS) served as control for the specificity of the siRNA knockdown. Despite 50–75% knockdown of basal USP14 protein levels ( **B**), no changes in Flag-α-synuclein expression were detected ( **C**).

Because there was a possibility that even the untagged-USP14 constructs were not functional, we tested whether siRNA knock down of endogenous USP14 would increase turnover of substrate. Lee *et al.* (2010) showed that *Usp14 ^-/-^* mouse embryonic fibroblasts had lower levels of tau or TDP-43 than those overexpressing wild-type USP14. Therefore, we reasoned that USP14 knockdown should result in lower levels of substrate. To avoid variability resulting from transient transfections, we tested USP14 knockdown in a stable Flag-tagged α-synuclein U2OS cell line. As shown in Figure 3, four different siRNAs (A58, A59, A60 and A90; 100nM) caused a 50–75% decrease in endogenous USP14 protein levels at 60 or 96 hours post-transfection ( Figure 3A, B). No changes in Flag-α-synuclein were detected ( Figure 3A, C).

**Figure 4.**
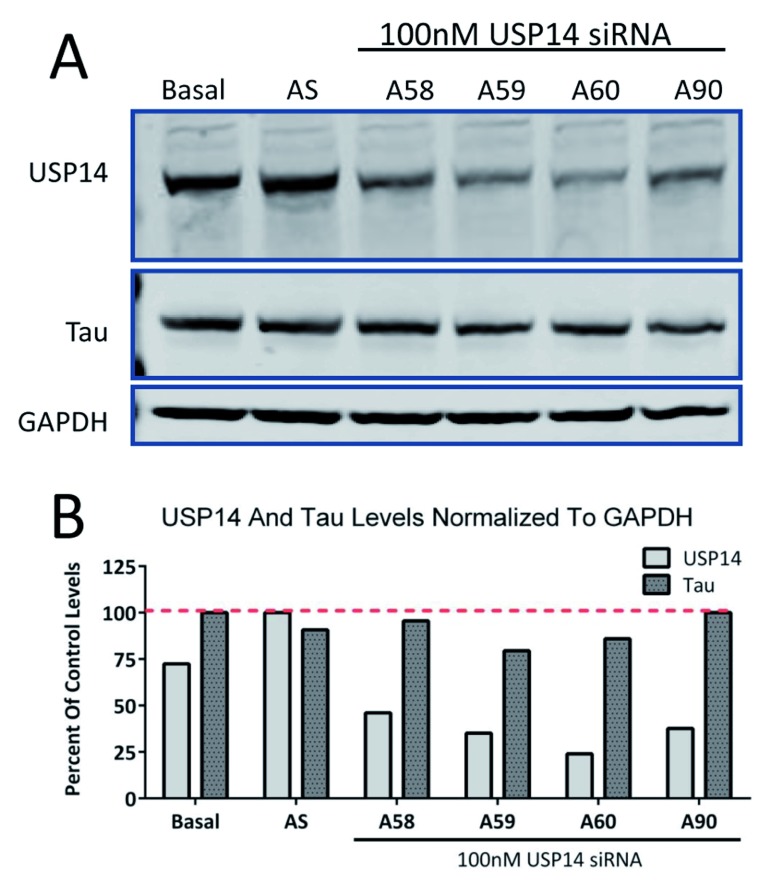
siRNA knockdown of endogenous USP14 does not decrease levels of endogenous tau in SH-SY5Y cells. SH-SY5Y cells endogenously expressing tau were transfected with 100nM USP14 siRNAs from Ambion (A58, A59, A60 or A90) or scrambled siRNA (AS) for 72 hours. Cells were lysed and analyzed by western blot using a standard protocol. A 50–75% decrease in USP14 protein levels was achieved compared to scrambled control, but no change in basal tau protein levels ( **A**, **B**).

Finally, to eliminate the concern that the artificial levels of the transiently or stably overexpressed substrates caused the lack of effect, we repeated the siRNA knockdown experiment in SH-SY5Y cells that endogenously express tau using siRNAs from Ambion (A58, A59, A60 and A90; 100nM). As shown in a representative western blot in Figure 4, no changes in endogenous tau levels were observed despite a 50–75% knockdown of endogenous USP14 protein levels. This experiment was repeated with four siRNAs from Qiagen at 48 hours and despite a 60–75% knockdown of USP14, we did not observe a consistent relationship between knockdown of USP14 and tau levels (not shown).

## Conclusions

Though we took several different approaches to assay the effects of USP14 on substrate levels, we were unable to confirm a robust role for USP14 in tau or TDP-43 degradation in our experimental systems. The possibility remains that differences in our methods (such as using a different expression vector) caused the discrepancies between our data and those in Lee *et al.* (2010). For example, the levels of proteasome-bound USP14 may have differed or protein synthesis and degradation rates may have been altered with our expression system. USP14 might also exert alternative functions that are dependent on substrate or cellular context: In a cellular model of prion disease, overexpression of catalytically inactive USP14 reduced accumulation of prion protein ( Homma *et al.*, 2015), whereas in a cellular Huntington’s disease model overexpression of catalytically inactive USP14 had no effect on huntingtin protein aggregates ( Hyrskyluoto *et al.*, 2014). Instead, overexpression of wild type USP14 reduced huntingtin aggregation. In USP14-deficient ax ^J^ mice *in vivo* Wilson and colleagues found no changes in endogenous tau or ataxin-3 protein levels, but did observe a difference in phosphorylated tau ( Jin *et al.*, 2012). They also generated mice expressing catalytically inactive USP14 and could not detect altered proteasomal function in these mice, although tau levels were not analyzed ( Vaden *et al.*, 2015). In combination, these studies highlight the complexity of USP14 biology and future research is needed to unravel the mechanisms that give rise to the apparent discrepancies. We hope our findings serve as a starting point for further discussion, collaboration, and research in this field.

## Data availability

The data referenced by this article are under copyright with the following copyright statement: Copyright: © 2016 Ortuno D et al.

Data associated with the article are available under the terms of the Creative Commons Zero "No rights reserved" data waiver (CC0 1.0 Public domain dedication). 

Open Science Framework: Dataset: Does inactivation of USP14 enhance degradation of proteasomal substrates that are associated with neurodegenerative diseases?, doi 10.17605/OSF.IO/7G3MJ ( Ortuno *et al.*, 2016).

## Figures and Tables

**Figure S1.**
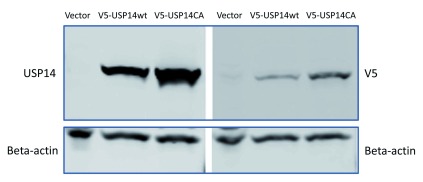
Validation of chicken polyclonal USP14 antibodies. 1μg V5-tagged USP14, V5-tagged USP14(CA) or empty vector control constructs were transfected in HEK293 cells and probed with V5 or chicken polyclonal anti-USP14 antibodies. Beta-actin served as loading control.
