# Supplementary material for: Does inactivation of USP14 enhance degradation of proteasomal substrates that are associated with neurodegenerative diseases?
Source: F1000Res. 2016 Apr 25;5:137. Originally published 2016 Feb 4. [Version 2] doi: 10.12688/f1000research.7800.2 (PMC4792207; doi:10.12688/f1000research.7800.2)
Supplement: Peer review history

# Peer review: Referee response for version 2

**DOI:** 10.5256/f1000research.9232.r12565

**Published:** 2016-05-06

**Authors:** Scott Wilson

**Affiliations:** 1 Department of Neurobiology, Evelyn F. McKnight Brain Institute, Civitan International Research Center, University of Alabama at Birmingham, Birmingham, AL, USA

Concerns on data quantitation have been addressed.  Previous comments on the stability of proteasome associated USP14 and determining protein stability by measuring steady state levels still remain.

I have read this submission. I believe that I have an appropriate level of expertise to confirm that it is of an acceptable scientific standard.

# Peer review: Referee response for version 2

**DOI:** 10.5256/f1000research.9232.r13707

**Published:** 2016-05-04

**Authors:** Scott T Brady

**Affiliations:** 1 Department of Anatomy and Cell Biology, University of Illinois at Chicago, Chicago, IL, USA

The changes are quite modest, but do address the minor points I raised. However, the remainder of the comments remain valid.

I have read this submission. I believe that I have an appropriate level of expertise to confirm that it is of an acceptable scientific standard.

# Peer review: Referee response for version 1

**DOI:** 10.5256/f1000research.8397.r12882

**Published:** 2016-03-14

**Authors:** Scott Wilson

**Affiliations:** 1 Department of Neurobiology, Evelyn F. McKnight Brain Institute, Civitan International Research Center, University of Alabama at Birmingham, Birmingham, AL, USA

Ubiquitin-dependent protein degradation involves the assembly of ubiquitin chains on specific proteins followed by their recognition and subsequent degradation by the proteasome. Since ubiquitination is a reversible reaction, there has been great interest in understanding the role of protein deubiquitination as a mechanism to regulate protein degradation. This is particularly true in post-mitotic neurons where lowering the burden of ubiquitinated aggregates of tau, Htt, alpha-synuclein and TDP-43 observed in Alzheimer’s Disease, Huntington’s Disease, Parkinson’s Disease and Amyotropic lateral sclerosis, respectively, is an attractive therapeutic intervention for the treatment of these diseases.

Ubiquitinated proteins stably associate with the proteasome through their interaction with proteasomal ubiquitin binding proteins. Following binding, and prior to degradation by the proteasome, ubiquitinated proteins are stripped of their ubiquitin tag. The disassembly of ubiquitin chains by proteasomal deubiquitinating enzymes serves multiple functions, including maintaining ubiquitin pools and determining whether a substrate will be released or degraded by the proteasome. The ubiquitin-specific protease 14 (USP14) is a proteasome-associated deubiquitinating enzyme that is required to maintain ubiquitin levels by preventing conjugated ubiquitin from entering the proteasome. Previous studies have also indicated that either a pharmacological block or genetic inactivation of USP14’s ubiquitin-hydrolase activity can reduce the steady-state levels of overexpressed aggregate-prone proteins tau, TDP-43 and ataxin-3 in immortalized cell lines. These findings suggested that blocking USP14’s deubiquitinating activity would lead to enhanced degradation of ubiquitinated substrates by preventing the substrates from being released by the proteasome prior to their commitment to degradation. 

Studies in mice provide support for an essential role for USP14 in controlling ubiquitin pools. Analysis of USP14-deficient mice revealed a significant loss of ubiquitin in multiple tissues, including the brain, and even greater loss of ubiquitin at synaptic terminals. Restoration of ubiquitin pools in these mice restored some of the synaptic transmission deficits caused by the loss of USP14, indicating a requirement for USP14 in ubiquitin homeostasis. Contrary to what was observed in immortalized cell lines, there was no detectable change in the steady state levels of the aggregate-prone proteins tau and ataxin-3 in the USP14-deficient mice.  However, increased levels of phosphorylated tau were observed in the USP14-deficient mice and correlated with elevated levels of activated JNK, ERK and AKT.  While USP14 still remains an interesting target for therapeutic intervention in protein-aggregate diseases, its role in controlling the degradation of specific proteins is not clear.

               

This study by Ortuno *et. al.* aims to further investigate a role for USP14 in controlling proteasomal degradation of aggregate-prone proteins. To do this, the authors first investigated if either overexpression of wild type USP14 or ubiquitin-hydrolase inactive USP14 would alter the levels of transfected tau or alpha-synuclein in an HEK293 cell line. If USP14 acts as an inhibitor of protein degradation, then overexpression of wild type USP14 should lead to increased levels of these aggregate-prone proteins while overexpression of ubiquitin-hydrolase inactive USP14 should reduce their levels.  However, increasing either wild type or ubiquitin hydrolase inactive USP14 did not result in a detectable change in the steady-state levels of tau or alpha-synuclein.The authors then investigated if lowering the levels of USP14 in the neuronal SHSY5Y cell line, which expresses endogenous tau, would result in decreased tau levels. Although the authors were able to significantly decrease the expression of USP14, they did not observe any significant change in the level of endogenous tau.  The authors were therefore unable to confirm a role for USP14 in controlling the degradation of aggregate-prone proteins.

The title is appropriate and the abstract provides a suitable summary. The experiments conducted in this study all generated high quality data and included appropriate controls. The authors provided a reasonable conclusion and potential reasons for differences between their results and those previously reported on USP14.

Concerns:

1. The entire premise of this paper is based on the manipulation of proteasome-bound USP14.  Unfortunately, the authors did not determine the level of proteasome-bound USP14. This is particularly important for the transfection of USP14(CA) and the *Usp14* siRNA knockdown experiments. If proteasome-bound USP14 has a long half-life, then these manipulations may not have significantly displaced endogenous USP14 from the proteasome.
2. The steady state level of any protein depends on the rates of synthesis and degradation.  However, the measurements in this report did not take into account either of these variables.  While highly unlikely, changes in protein turnover due to manipulation of USP14 may have caused increased turnover of tau, alpha-synuclein or TDP-43 with a corresponding increase in synthesis, resulting in no change in protein abundance.
3.  There are no error bars in figures 1, 2 and 4. 
4. It is not clear if the quantitations represent averages from replicate immunoblots or if a single blot was performed for each experiment.

I have read this submission. I believe that I have an appropriate level of expertise to confirm that it is of an acceptable scientific standard, however I have significant reservations, as outlined above.

# Peer review: Referee response for version 1

**DOI:** 10.5256/f1000research.8397.r12829

**Published:** 2016-03-09

**Authors:** Thomas Kodadek

**Affiliations:** 1 Department of Cancer Biology, Scripps Research Institute - Florida, Jupiter, FL, USA

This study tests the previously published assertion that reducing the activity of USP14, a proteasome-associated deubiqtuitylase, results in decreased levels of neurotoxic proteins such as TDP43 and Tau. The bottom line of the study is that manipulation of USP14 level and activity in a cell model system has no discernable effect of the levels of these proteins, contrary to expectations based on a previously published 2010 study ^1^. While this is obviously a model system with unknown relevance to *bona fide *neurons *in vivo*, the experiments appear to be well done and the data support the conclusions. The authors are careful to point out that there are minor differences between some of their protocols and those used in the 2010 study and call for increased communication and collaboration between interested laboratories to determine if USP14 is truly a good drug target for neurodegenerative diseases. This is entirely appropriate.

I have read this submission. I believe that I have an appropriate level of expertise to confirm that it is of an acceptable scientific standard.

## References

[1] LeeBHLeeMJParkSOhDCElsasserSChenPCGartnerCDimovaNHannaJGygiSPWilsonSMKingRWFinleyD: Enhancement of proteasome activity by a small-molecule inhibitor of USP14. *Nature*.2010;467(7312) : 10.1038/nature09299 179-84 10.1038/nature09299 20829789PMC2939003

# Response

**Published:** 2016-03-11

**Authors:** Thomas Kodadek

**Affiliations:** Department of Chemistry and Cancer Biology, The Scripps Research Institute, Scripps Florida, USA

Since posting my review of this F1000Research article, a colleague made me aware of two manuscripts that are highly relevant to this topic, but were not cited by Ortuno *et al.*

They are:

Homma T, Ishibashi D, Nakagaki T, *et al.*: Ubiquitin-specific protease 14 modulates degradation of cellular prion protein. *Sci Rep*. 2015; **5**:11028.

McKinnon C, Goold R, Andre R *, et al*.: Prion-mediated neurodegeneration is associated with early impairment of the ubiquitin-proteasome system. *Acta Neuropathol*. 2016; **131**(3): 411-425.

Both of these papers report that levels of prion proteins in neurons are strongly influenced by USP14 activity. 

Thus, while the experiments conducted by Ortuno *et al*. reported in this communication do not seem to indicate that manipulation of USP14 has a major effect on TDP-43 and α-synuclein levels under their conditions, the major findings of the 2010 Nature paper by Lee *et al. *are strongly supported by these two studies. Thus, these papers should have been cited by Ortuno *et al*. in their F1000Research article.  I apologize for not being aware of these two studies when I reviewed this work.

# Peer review: Referee response for version 1

**DOI:** 10.5256/f1000research.8397.r12772

**Published:** 2016-03-07

**Authors:** Scott T Brady

**Affiliations:** 1 Department of Anatomy and Cell Biology, University of Illinois at Chicago, Chicago, IL, USA

Many adult-onset neurodegenerative diseases are associated with aggregates of misfolded proteins or peptides. A number of groups have proposed that those aggregates that are intracellular, such as tau, synuclein and TDP-43, may result from defects in the protein-degradation pathways like the proteasome which slows normal protein turnover. Such proposals lead naturally to the idea that enhancing endogenous protein degradation pathways is a potential therapeutic strategy to reduce aggregate levels, thereby slowing or blocking disease progression. This manuscript focuses on USP14, a deubiquinating enzyme associated with the proteasome that catalyzes the release of ubiquitin for proteins targeted for degradation and allow the ubiquitin to be recycled for targeting other proteins to the proteasome. Recycling of ubiquitin is particularly important in domains far removed from sites where newly synthesized ubiquitin is available. The need to transport ubiquitin to synaptic terminals is an obvious example. Studies in a mouse model deficient in USP14 found reduced tissue ubiquitin levels in all tissues with a particularly significant loss in synaptic terminals and there is evidence of altered synaptic transmission in these mice. Curiously, there were no obvious accumulations of specific proteins or increased aggregates in brains noted in descriptions of this USP14-deficient mouse, despite its putative role in proteasome function. Subsequent studies failed to show a difference in endogenous levels of tau in the USP14-deficient mouse and a second mouse line expressing a catalytically dead USP14 did not find altered proteasomal activity.

Understanding the role of USP14 in clearance of pathogenic proteins is complicated by the fact that different proteins may involve different actions of USP14. For example, degradation of some proteins is normal in the presence of catalytically dead USP14, while others require catalytic activity. Tau and TDP-43 were both reported to be in the latter category as recombinant proteins accumulated to a higher level when expressed with wild type, but not with catalytically inactive USP14. This finding was the basis for suggestions that inhibition of USP14 might enhance clearance of these proteins.

Experiments described in this report sought to further characterize the ability of USP14 to modulate the clearance of tau, TDP-43 and α-synuclein. Unfortunately, increased levels of either wild type or catalytically inactive USP14 had no effect on levels of tau or TDP-43 and siRNA knockdown of endogenous USP14 failed to affect cellular levels of α-synuclein or alter endogenous expression of tau protein in a differentiated neuroblastoma cell line. A variety of different approaches to alter levels of USP14 failed to confirm the previous reports of altered clearance. The experiments are carefully documented and well controlled, suggesting that USP14 does not play a role in modulating the clearance of these proteins, consistent with the mouse studies. The conclusion is that inhibition of USP14 is not a promising therapeutic target for enhancing clearance of pathogenic proteins in adult-onset neurodegeneration. Although these cell-based assays make a strong case for this conclusion, data from the mouse models were never consistent with this proposal, since they showed no obvious evidence of proteasomal dysfunction or reduced tau levels. Indeed, given the fact that loss of USP14 catalytic activity in the mouse led to defects in synaptic transmission, it is hard to see how inhibition of USP14 was a plausible therapeutic strategy.

Minor Points.

1. The quantitative data in figures 1 and 2 are expressed as being normalized to beta actin levels. No indication is given as to the number of replicates or whether any statistical analysis was done. The raw data is shown as immunoblots with epitope tags, so the conclusions appear justifiable. Nevertheless, the number of experimental replicates must be given and the case would be more compelling with statistics. 
2. Technically, the bar graphs show ratios, which are dimensionless, not arbitrary units.

I have read this submission. I believe that I have an appropriate level of expertise to confirm that it is of an acceptable scientific standard, however I have significant reservations, as outlined above.
